# TEVAR-Only Management of Abdominal Aortic Aneurysm Rupture Caused by Acute Type B Aortic Dissection after EVAR: A Case Report

**DOI:** 10.3400/avd.cr.26-00009

**Published:** 2026-04-11

**Authors:** Toshinori Takahashi, Osamu Namura, Shinya Mimura

**Affiliations:** Department of Cardiovascular Surgery, Niigata Prefectural Central Hospital, Joetsu, Niigata, Japan

**Keywords:** type B aortic dissection, ruptured abdominal aortic aneurysm, EVAR, TEVAR

## Abstract

Type B aortic dissection after endovascular aneurysm repair (EVAR) is a rare late complication and may cause rupture of a previously stable abdominal aortic aneurysm (AAA). A 79-year-old male developed acute Stanford type B aortic dissection 12 years after EVAR, with distal extension into an infrarenal AAA resulting in rupture and circulatory collapse. A large primary entry tear was located in the descending thoracic aorta. Emergency thoracic endovascular aortic repair (TEVAR) was performed to seal the entry tear, achieving rapid hemodynamic stabilization without abdominal intervention. This case suggests that AAA rupture secondary to acute type B aortic dissection after EVAR can be managed with TEVAR alone in selected acute settings.

## Introduction

Acute type B aortic dissection is often managed medically during the acute phase, as conservative treatment is associated with a relatively low early mortality rate of approximately 10%. However, emergency surgical intervention is indicated for complicated cases presenting with organ malperfusion, rupture, or rapid expansion. Rupture occurs in 9.6% of acute type B aortic dissections; however, despite recent advances in surgical and endovascular management, the in-hospital mortality remains high at 42.4%, indicating that it remains a life-threatening condition.^1)^

We report a rare case of acute type B aortic dissection that developed after endovascular aneurysm repair (EVAR). The dissection extended distally to involve a preexisting abdominal aortic aneurysm (AAA), which subsequently ruptured. The patient was successfully treated with emergency thoracic endovascular aortic repair (TEVAR).

## Case Report

A 79-year-old male with a history of hypertension and bladder cancer had undergone EVAR with a Zenith Flex stent graft (Cook Medical, Bloomington, IN, USA) at our institution 12 years earlier for an AAA measuring 58 mm in maximum diameter. Annual follow-up imaging showed no evidence of endoleak, and the aneurysm gradually decreased in size to 41 mm.

**Figure 1:**
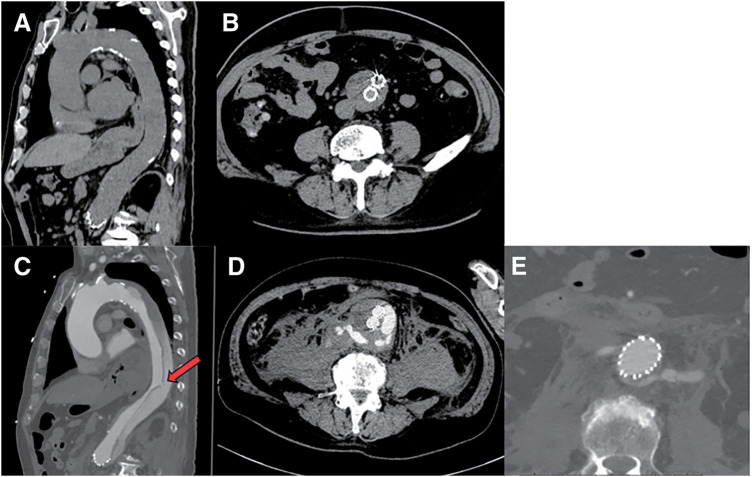
Fig. 1 Serial CT findings before and after onset of type B aortic dissection. (**A**, **B**) CT at the initial emergency department visit. The AAA showed a tendency toward reduction, and no clear findings suggestive of aortic dissection were observed. (**C**) CT at representation demonstrated an acute Stanford type B aortic dissection with a large primary entry tear located in the descending thoracic aorta (arrow). The false lumen extended from the origin of the left subclavian artery to the infrarenal AAA. (**D**) A retroperitoneal hematoma adjacent to the AAA indicated rupture of the false lumen within the aneurysm. (**E**) A suspected re-entry tear was identified at the level of the left renal artery. CT: computed tomography; AAA: abdominal aortic aneurysm

He presented to the emergency department with low back pain. At that time, he was hemodynamically stable. Laboratory tests revealed a hemoglobin level of 11.7 g/dL and a D-dimer level of 6.4 μg/mL with no significant change compared with the previous outpatient evaluation. Non-contrast computed tomography (CT) demonstrated no apparent findings suggestive of acute aortic dissection or aneurysmal rupture (**Fig. 1A** and **1B**). As there were no clinical or imaging findings strongly indicative of acute aortic pathology, he was discharged home the same day. The following morning, he returned with similar complaints, received symptomatic treatment, and was again discharged after no significant interval change was noted. Four hours later, he re-presented with worsening back pain and vomiting. His blood pressure was 126/77 mmHg, and he was tachycardic with a heart rate of 100 beats per minute (bpm). Repeat laboratory tests demonstrated a decline in hemoglobin to 9.6 g/dL and a marked elevation of D-dimer to 53.6 μg/mL. These findings raised a strong suspicion of acute aortic pathology, prompting contrast-enhanced CT, which demonstrated an acute type B aortic dissection.

The false lumen was patent and extended from the origin of the left subclavian artery to the previously repaired AAA. The primary entry tear was located in the descending thoracic aorta just above the diaphragm, and a possible re-entry site was noted around the left renal artery. CT also showed enlargement of the AAA measuring 58 mm and retroperitoneal hematoma, suggesting rupture of the false lumen at that site (**Fig. 1C**–**1E**). Soon after the CT, the patient developed hypotension followed by pulseless electrical activity. Cardiopulmonary resuscitation was initiated, and return of spontaneous circulation was achieved after intravenous administration of adrenaline. The patient was subsequently intubated and placed on mechanical ventilation.

**Figure 2:**
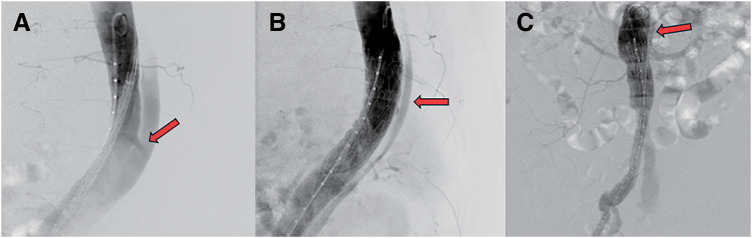
Fig. 2 Intraoperative angiography and TEVAR procedure. (**A**) Intraoperative angiography of the descending thoracic aorta demonstrated a large primary entry tear with contrast flow into the false lumen (arrow). A thoracic stent graft was deployed to suppress false-lumen perfusion. (**B**) Angiography of the same region after stent graft deployment revealed residual retrograde flow into the false lumen from the distal side (arrow). (**C**) Selective angiography of the abdominal visceral arteries demonstrated a re-entry tear near the left renal artery (arrow), consistent with the CT findings. No contrast flow into the AAA was observed. TEVAR: thoracic endovascular aortic repair; CT: computed tomography; AAA: abdominal aortic aneurysm

Given that closure of the entry site could reduce false-lumen flow and achieve hemostasis, emergency TEVAR was performed (**Fig. 2**). Angiography confirmed a large entry tear in the descending aorta, consistent with the CT findings, and a CTAG stent graft (W. L. Gore & Associates, Newark, DE, USA) was deployed at that site (**Supplementary Video 1**). Post-deployment angiography revealed persistent retrograde flow into the false lumen from the distal side (**Supplementary Video 2**). Additional angiography around the visceral branches showed contrast entering the proximal false lumen through the re-entry near the left renal artery, but no further extravasation into the aneurysm (**Supplementary Video 3**). As the hemodynamics stabilized at this point, the procedure was concluded.

The patient was admitted to the intensive care unit under sedation and mechanical ventilation. CT performed on postoperative day 2 demonstrated no enlargement of the AAA and a reduction of the retroperitoneal hematoma. His level of consciousness did not improve, and brain magnetic resonance imaging (MRI) on postoperative day 8 revealed multiple acute watershed infarctions on diffusion-weighted imaging, consistent with hypoperfusion injury after preoperative circulatory collapse. Carotid imaging demonstrated approximately ≥50% stenosis at the origins of both internal carotid arteries by visual estimation. The patient was successfully extubated on postoperative day 20 without requiring tracheostomy and transferred to a general ward on day 21. After continued rehabilitation, surgical gastrostomy was performed on postoperative day 74. He was eventually transferred to a long-term care facility 6 months postoperatively with strong family support.

**Figure 3:**
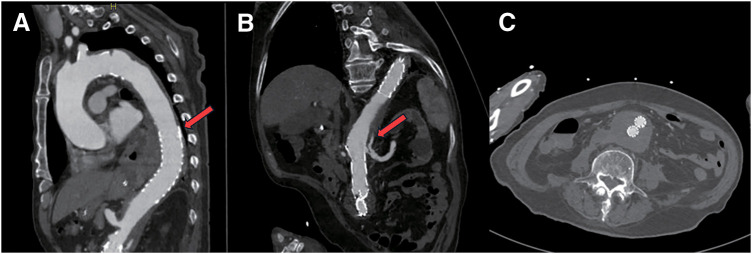
Fig. 3 Postoperative CT demonstrating false-lumen thrombosis. (**A**) Postoperative contrast-enhanced CT demonstrated successful TEVAR with complete closure of the primary thoracic entry tear (arrow). (**B**) Although a re-entry tear at the level of the renal artery persisted (arrow), only minimal residual contrast enhancement within the false lumen was observed, indicating progressive thrombosis. (**C**) The infrarenal abdominal aortic aneurysm had decreased in size, and the retroperitoneal hematoma showed signs of resolution. CT: computed tomography; TEVAR: thoracic endovascular aortic repair

Follow-up contrast-enhanced CT performed 2 months after the procedure demonstrated satisfactory thrombosis of the false lumen, with only minimal contrast enhancement in the proximal portion near the renal arteries. The AAA had decreased in size, and the retroperitoneal hematoma showed signs of resorption (**Fig. 3**). At 1-year follow-up, the AAA diameter had further decreased to 37 mm, and no additional intervention has been required to date.

## Discussion

Acute type B aortic dissection occurring after EVAR is an uncommon but clinically significant complication that may arise in both the early and late postoperative periods. Although most acute type B dissections can be managed conservatively, the presence of complications such as malperfusion or rupture dramatically worsens prognosis and necessitates urgent intervention. Among these, rupture involving a preexisting AAA after EVAR represents a particularly catastrophic and rare scenario, in which survival is generally unlikely without prompt surgical intervention.^2–4)^

In the present case, acute type B aortic dissection developed more than a decade after EVAR and extended distally into a previously stable infrarenal AAA, ultimately resulting in rupture and circulatory collapse. The unusually long interval between EVAR and dissection onset, together with the presence of a large primary entry tear located in the descending thoracic aorta rather than at the stent graft edges, suggests a spontaneous dissection rather than an immediate device-related complication.

The most notable aspect of this case is that definitive hemostasis was achieved by TEVAR alone, without any direct abdominal intervention. By sealing the primary thoracic entry site, TEVAR was expected to depressurize the false lumen throughout the thoracoabdominal aorta rapidly and thereby achieve indirect hemostasis at the rupture site. In contrast, all previously reported cases of AAA rupture secondary to type B aortic dissection after EVAR required open abdominal surgical repair, either alone or in combination with additional procedures.^5–8)^ The present case demonstrates that TEVAR alone may achieve hemostasis in selected patients, particularly those with profound hemodynamic instability in whom open surgery carries substantial risk.

In the present case, although retrograde flow into the false lumen from a suspected re-entry near the left renal artery was observed angiographically, no further extravasation into the aneurysm sac was detected after TEVAR, and hemodynamics stabilized promptly. This finding suggests that the thoracic entry tear was the dominant hemodynamic driver of rupture. A previous report described a staged endovascular strategy in which residual re-entry sites of type B aortic dissection were subsequently closed by additional endovascular intervention after initial stabilization.^9)^ In an acute and hemodynamically unstable setting, such as the present case, similar adjunctive procedures might also have been feasible as an emergency, single-stage endovascular approach if hemostasis had not been achieved by TEVAR alone.

Some authors have argued that TEVAR may be insufficient in cases where distal re-entry sites maintain false-lumen perfusion, advocating early open surgical repair.^5)^ Although this concern is valid, open surgery in the acute phase of dissection carries substantial risk due to aortic wall fragility and the potential for uncontrollable bleeding.

Chait et al. described a hybrid strategy in which TEVAR was performed initially, followed by infrarenal aortic banding when persistent false-lumen pressurization was observed.^6)^ This banding technique avoids direct manipulation of the fragile dissected aortic wall, thereby potentially reducing bleeding risk in the acute phase. Additional interventions have also been reported in cases with hemodynamic deterioration after initial treatment.^8)^ These findings indicate that persistent false-lumen flow may require further procedures beyond proximal entry closure.

In contrast, in the present case, neither persistent false-lumen perfusion on completion angiography nor aneurysmal enlargement on early postoperative CT was observed, and hemodynamics stabilized promptly after TEVAR alone. These findings suggest that closure of the primary thoracic entry tear was sufficient to reduce false-lumen pressurization and achieve indirect hemostasis at the rupture site. Nevertheless, distal re-entry flow may sustain pressurization even after successful proximal entry closure, and secondary intervention should be considered when persistent perfusion, progressive aneurysmal enlargement, or ongoing hemodynamic instability is present. Careful postoperative surveillance remains essential.

Finally, this case highlights the importance of lifelong and comprehensive surveillance after EVAR. Even when the infrarenal aneurysm remains stable for many years without evidence of endoleak, late-onset thoracic aortic events may precipitate fatal abdominal complications. Clinicians should therefore maintain a high index of suspicion for acute aortic dissection in EVAR patients presenting with back or abdominal pain, regardless of the elapsed time since the initial procedure.

## Conclusion

Abdominal aortic aneurysm rupture following acute type B aortic dissection after EVAR represents a rare but highly lethal condition. This case demonstrates that, when rupture is driven by thoracic false-lumen pressurization, TEVAR alone can serve as a definitive, life-saving treatment. Prompt recognition of the underlying pathophysiology and rapid endovascular intervention are essential for achieving survival in this critical setting.

## Supplementary Materials

Supplementary Video 1 Intraoperative angiography of the descending thoracic aorta identified the primary entry tear, and a 31–26 mm × 10 cm CTAG stent graft (W. L. Gore & Associates) was deployed to suppress false-lumen perfusion.

Supplementary Video 2 Contrast angiography after stent graft deployment confirmed closure of the primary entry tear; however, retrograde perfusion of the false lumen from a distal re-entry site was observed.

Supplementary Video 3 Angiography distal to the deployed stent graft showed retrograde opacification of the false lumen via a re-entry near the left renal artery, with no contrast inflow into the false lumen of the AAA.
